# Efficiency of Feed Utilization, Ruminal Traits, and Blood Parameters of Goats Given a Total Mixed Diet Ration Containing Extracted Oil Palm Meal

**DOI:** 10.3390/vetsci9110612

**Published:** 2022-11-04

**Authors:** Pin Chanjula, Sarong So, Chanon Suntara, Rittikeard Prachumchai, Anusorn Cherdthong

**Affiliations:** 1 Animal Production Innovation and Management Division, Faculty of Natural Resources, Hat Yai Campus, Prince of Songkla University, Songkhla 90110, Thailand; 2 Department of Animal Science, Faculty of Agriculture and Food Processing, National University of Battambang, Battambang 02352, Cambodia; 3 Tropical Feed Resource Research and Development Center (TROFREC), Department of Animal Science, Faculty of Agriculture, Khon Kaen University, Khon Kaen 40002, Thailand

**Keywords:** low-fat oil palm meal, digestibility, globulin, triglyceride, volatile fatty acid

## Abstract

**Simple Summary:**

Animal production in the tropics has relied heavily on agro-industrial waste products as an alternative to expensive protein and energy sources and as a means of lowering production costs. After extraction, low-fat oil palm meal is created as a byproduct of biodiesel and may be utilized as an alternate feed source for animals. The inclusion of extracted oil palm meal in the diet of goats with up to 20% DM was shown to be beneficial.

**Abstract:**

This study aimed to evaluate the inclusion levels of extracted oil palm meal (EOPM) from biodiesel byproducts in the total mixed ration (TMR) for goats on feed intake, nutrient digestibility, nitrogen balance, blood parameters, and rumen fermentation characteristics. The EOPM was included at 0%, 10%, 20%, and 30% dry matter (DM) to formulate into four dietary treatments and assigned to goats with an average age of 12 months and an initial body weight of 23 ± 0.5 kg in a 4 × 4 Latin square design. The PROC MIXED procedure was used to analyze all data, and the orthogonal polynomial was tested for EOPM levels using the SAS program. The DM intake, nitrogen, and metabolizable energy linearly (*p* < 0.05) increased when there was increased EOPM inclusion. Increasing EOPM inclusions resulted in (*p* < 0.05) a linear increase in fiber digestibility but did not affect DM, crude protein, or ether extract. Total volatile fatty acids increased (*p* < 0.05) linearly with increasing EOPM inclusions. The EOPM inclusions did not alter the bacteria, fungi, and protozoal populations. Blood parameters were not affected by the inclusions of EOPM except globulin, triglyceride, and MCV concentration. This study revealed that EOPM could be included in up to 20% DM in TMR for goats with no negative impact.

## 1. Introduction

Agro-industrial byproducts have been playing critical roles in tropical animal production with regard to solving high-cost protein and energy ingredients and reducing production costs. Some of these byproducts are byproducts from the palm oil production industry, including oil palm frond, palm oil decanter cake, oil palm meal (OPM), and palm kernel meal [1,2,3]. The OPM is produced during oil refinement from palm fruit, which is composed of a husk shell and kernel cake containing 8% crude protein (CP) and 7.4% fat [2], and 30% crude fiber [4], which are available in large amounts, at low cost, and suitable as alternative feed resources for ruminants. In dairy cows, the inclusion of OPM up to 20% of dry matter (DM) in the ration decreased the protozoal population, which is caused by the fat content in OPM, but decreased DM intake and digestibility of DM, organic matter (OM), and CP [2]. High-fat content in OPM after mechanical extraction using a screw press limits its inclusion rate in a diet and decreases its storage shelf-life. Solvent extraction can be an alternative way of removing fat content, and regarding oil extraction with solvent, hexane is more effective than ethanol [5]. However, toxic contaminants with hexane residual in ruminant feeds after extraction need to be paid attention to. Thus, ethanol is superior to hexane, easy to remove, and safer than hexane residue [6]. Somnuk et al. [7] reported that ethanol extraction could remove 17.4 wt.% of oil from 5000 g of OPM, and low-fat oil palm meal (EOPM) after extraction is produced as a biodiesel byproduct and can be used as an alternative feed resource for animals.

Based on our review, the inclusion of EOPM in ruminant diets has not yet been studied. The hypothesis can be made that EOPM could be added higher at more than 20% DM of a diet without a negative effect on intake and digestibility. Therefore, this study aimed to evaluate the inclusion levels of EOPM in the total mixed ration for goats on feed intake, nutrient digestibility, nitrogen balance, blood parameters, and rumen fermentation characteristics.

## 2. Materials and Methods

### 2.1. Extracted Oil Palm Meal (EOPM)

The EOPM was provided by the Department of Mechanical Engineering, Faculty of Engineering, Prince of Songkla University, Thailand. Briefly, OPM was extracted with 95 volume % hydrous ethanol at a ratio of 1:1 (*w*/*w*) under a constant mixing of 300 rpm at 30 °C for 11 min [7]. After extraction, EOPM was collected to analyze the chemical compositions. The chemical composition of OPM and EOPM are provided in Table 1.

### 2.2. Goats, Experimental Diets, and Feeding Procedures

The experiment was conducted at the experimental farm at the Prince of Songkla University, Hat Yai, Songkhla, Thailand. Goats were used and taken care of under the approval number ([48], 2017) issued by the Prince of Songkla University Animal Care and Use Committee.

Four male crossbreds, 50% Anglo-Nubian, with an average age of 12 months and an initial body weight (BW) of 23 ± 0.5 kg were randomly assigned in a 4 × 4 Latin Square Design. Goats were kept individually in pens (0.115 × 0.95 m) under well-ventilated sheds where water and mineral blocks were available at all times. Levels of EOPM at 0%, 10%, 20%, and 30% based on DM were added in concentrate to form four dietary treatments. The experiment lasted for four periods, and each period was divided into fourteen days for feed adaptation and seven days for data collection. Goats were fed total mixed ration (TMR) diets at a ratio of 75% concentrate and 25% oil palm fronds as a sole roughage feed. The TMR diets were formulated as isonitrogenous at 15% CP on DM and an isocaloric of 4 kcal/kg DM according to the recommendation of NRC [8]. The ingredients of TMR and the chemical composition of TMR and EOPM are provided in Table 1. TMR diets were provided ad libitum at 8:00 a.m. and 4:00 p.m. with expected at least 10% orts. All the animals were housed in individual pens. Clean, fresh water and feed blocks were available at all times for the whole experiment. Goats were weighed at the beginning of the experiment and at the end of each period to adjust the feed DM supply.

### 2.3. Sample Collection and Laboratory Assay

The TMR feeding was recorded daily by weighing the offered and refused feeds in the morning feeding to calculate DM intake. Samples (300 g) of TMR and fecal samples were collected for seven days of each period and pooled by goats. To prevent nitrogen loss, urine was collected using a glass bottle containing 1 M of sulfuric acid for seven days during data collection period. On the last day of each period, samples (100 mL) of ruminal fluid were collected from each goat 0 h before feeding and 4 h after feeding using a stomach tube attached to a vacuum machine, with approximately 300 mL of ruminal fluid discarded first to avoid saliva contamination. A sample (3 mL) of blood was withdrawn at 0 h pre-feeding and 4 h post-feeding from each goat at the jugular vein position and kept in a test tube containing ethylenediaminetetraacetic acid to prevent blood coagulation.

Samples from TMR and feces were oven-dried at 60 °C for 48 h, ground through a 1 mm screen, and then analyzed for DM, ash, ether extract (EE), and nitrogen content according to AOAC [9]. The CP content was calculated by multiplying the nitrogen content by 6.25. Neutral detergent fiber (NDF) and acid detergent fiber (ADF) were analyzed using an Ankom fiber analyzer according to Van Soest et al. [10]. Urine samples were analyzed for nitrogen content according to AOAC [9]. Gross energy, feces, and urine were analyzed using a bomb calorimeter. The nutrient content in TMR and feces was used to study the digestibility. Nitrogen utilization was studied from the nitrogen content in TMR, feces, and urine. Metabolizable energy intake was estimated according to Kearl [11] using the equation of 1 kg DOM = 3.8 Mcal ME/kg.

The pH of the ruminal fluid was measured immediately after sampling using a glass electron pH meter (HANNA Instruments HI 98153 microcomputer pH meter, Singapore). After that, the sample of rumen fluid was separated into two parts. The first sample (45 mL) from 100 mL of ruminal fluid was kept in 9 mL of 1 M H_2_SO_4_, centrifuged at 3000× *g* for 15 min at 10 °C, and used to analyze ammonia nitrogen (NH_3_-N) concentration using the Kjeltech Auto 220 Analyzer (Foss, Runcorn, UK) according to Bremner and Keeney [12] and volatile fatty acid (VFA) such as acetic acid (C2), propionic acid (C3), butyric acid (C4) using high-performance liquid chromatography (Instruments by controller water 510 pumps (Millipore, TX, USA), UV Detector 210 nm., ODS reverse phase column (5 µ, 40 × 250 mm)) according to Samuel et al. [13]. Another sample (1 mL) from 100 mL of ruminal fluid was kept in 9 mL of 10% formaldehyde and used to enumerate the bacteria, protozoa, and fungi populations using a 400-groove hemocytometer (width × length × height = 1 × 1 × 0.1 mm) according to Galyean [14] using a microscope (Olympus BX51TRF, No. 2B04492, Olympus optical Co. Ltd., Tokyo, Japan).

Blood samples were centrifuged at 3000× *g* for 10 min to obtain plasma and sent to Stanbio Laboratory (An EKF Diagnostics Company, Boerne, TX, USA) to analyze blood urea nitrogen (BUN), PCV, blood glucose, cholesterol, triglyceride, albumin, globulin, WBC, RBC, HGB, and MCV using standard commercial kits in a fully automatic biochemical analyzer (HT82-BTS-330, Xihuavi Technology Co., Ltd., Beijing, China).

### 2.4. Statistical Analysis

Data from intake and digestibility were analyzed using the PROC MIXED procedure of SAS according to the model: Y_ijk_ = μ + B_i_(A_l_) + C_k_(A_l_) + D_j_ + ε_ijk_, where Y_ijk_ is each observation for a given variable, μ is the overall mean, Bi(A_l_) is the random effect of period i within square; C_k_(A_l_) is the random effect of goat k within square; D_j_ is the fixed effect of treatment and ε_ijk_ is the residual random term.

Data from rumen fermentation and blood parameters were analyzed using the PROC MIXED procedure of SAS according to the model: Y_ijkm_ = μ + B_i_(A_l_) + C_k_(A_l_) + t_m_ + D_j_ + ε_ijkm_ where Y_ijkm_ is each observation for a given variable, μ is the overall mean, B_i_(A_l_) is the random effect of period i within square l; C_k_(A_l_) is the random effect of goat k within square l; t_m_ is the random effect of sampling time at 0 h pre- and 4 h post-feeding; D_j_ is the fixed effect of treatment and ε_ijkm_ is the residual random term. Treatment means were compared using Duncan’s New Multiple Range Test. An orthogonal polynomial was used to study the trend effect of EOPM addition.

## 3. Result and Discussion

### 3.1. Voluntary Feed Intake and Nutrient Intake

Total dry matter (DM) intake (kg/d, %BW, and g/kg W^0.75^) linearly increased (L, P = 0.01) when increasing levels of EOPM were observed (Table 2). Inclusion of EOPM at 20% DM and 30% DM significantly increased total DM intake when compared to the control (0% EOPM). This could be due to the decrease in ether extract and gross energy in the diets causing animals to consume more feed to meet energy requirements [15]. In addition, total DM intake was not significant between 10%, 20%, and 30% DM of inclusions. Furthermore, dietary intake can be modified by fat content greater than 5% in the diet. Our finding was in contrast to the finding of Lunsin [2] who found a decrease in DM intake when increased inclusion of OPM was up to 30% DM, that caused by the increase in fat content over 5% DM in the diet. Increasing EOPM inclusions linearly (*p* < 0.05) increased the intake of OM, NDF, and ADF. The intake of OM, NDF, and ADF was significantly higher in 20% and 30% of EOPM when compared with the control. The increase in the intake of OM, NDF, and ADF could be related to the increase in DM intake. In contrast, Lunsin [2] found that increasing OPM inclusions significantly decreased the intake of OM, NDF, and ADF while increasing the fat intake. Decreasing the intake of OM, NDF, and ADF was related to the decrease in DM intake, while an increase in fat intake was due to the increase in fat content in the diet when OPM inclusions were increased. In addition, Castro et al. [16] increased palm kernel oil from 0% to 5.2% DM in diets, resulting in increased fat content in diets and subsequently decreased the intake of DM, OM, CP, fat, NDF, and ADF.

### 3.2. Digestibility and Intake of Nutrient Digestibility

The apparent digestibility of DM, OM, CP, and EE were similar (*p* > 0.05) among treatments (Table 3). However, apparent NDF and ADF digestibility increased (*p* < 0.05) when EOPM inclusion was increased. This may be attributed to the low-fat content in diets with higher EOPM levels and greater DM intake, whereas fat content greater than 5% in the diets may decrease digestion and bacterial growth [17,18,19,20,21]. Lunsin [2] fed oil palm meal up to 30% DM to dairy cow, resulting in a decrease in nutrient intake and digestibility. The author stated that the decrease in nutrient intake and digestibility was due to the high-fat content in oil palm meal and diets. However, not all studies found a decrease in nutrient digestibility when fat content was increased in diets. Castro et al. [16] found that increasing palm kernel oil from 0% to 5.2% DM in diets fed to finishing lambs significantly increased nutrient digestibility while significantly decreasing feed and nutrient intake, but the authors did not trace any reasons for the positive effect on nutrient digestibility when increasing palm kernel oil levels in diets. Santos et al. [22] fed palm kernel cake containing 12.2% of ether extract to lambs up to 30% DM in diets, increasing fiber digestibility, which is related to the increase in DM and fiber intake, while Chanjula et al. [1] found that feeding palm kernel cake up to 45% DM in diets decreased fiber digestibility in goats, which is related to the decrease in DM intake.

### 3.3. Ruminal Fermentation and Blood Urea Nitrogen

The effects of EOPM inclusions at 0, 10, 20, and 30% on ruminal pH, temperature, ammonia nitrogen (NH_3_-N), and blood urea nitrogen (BUN) are shown in Table 4. The mean ruminal temperature between 0 and 4 h after feeding was not affected by treatments and was 39.10 to 39.35 °C, which was stable and optimal for rumen microbe function [23,24,25]. The ruminal pH was found to be unaffected even up to a 30% EOPM dietary inclusion level. This is similar to the findings of Chanjula et al. [1], who used oil palm kernel pulp (PKC) at the levels of 15, 25, 35, 45, and 55%, respectively, in concentrate feed for goats. There was no change in ruminal pH between treatments (*p* > 0.05). The average pH did not change (6.22 to 6.53), which is an acceptable level (6.0 to 7.0) for the function of fiber-decomposing microorganisms, protein digestion, and cellulolytic bacteria [26,27,28]. The rumen NH_3_-N concentration was not different (*p* > 0.05), whereas NH_3_-N ranged from 20.53 to 21.79 mg/dL. Lunsin et al. [2] added that adding oil palm meal up to 40% DM in diets for lactating cows did not affect ruminal pH, temperature, and NH_3_-N concentration. There were no effects on blood urea nitrogen (BUN) at 0 and 4 h after feeding with EOPM inclusions. The BUN value ranged from 23.03 to 24.55 mg/dL. Similarly, Lewis [29] reported that the optimal BUN level of goats ranges from 11.2 to 27.7 mg/dL. The BUN concentrations vary by several factors, including age, nutrition, and protein consumption, in particular, the concentration of ruminal NH_3_-N. BUN levels were found to be significantly related to digestible protein consumption and to the concentration of ammonia produced in the rumen [30,31]. Lunsin [2] found that increasing oil palm meal from 0 to 40% DM in diets resulted in lower BUN concentrations in lactating cows, although this was not significantly different.

### 3.4. Blood Metabolites

The effects of EOPM inclusions at 0, 10, 20, and 30% on blood glucose, PCV, cholesterol, albumin, globulin, WBC, RBC, HGB, and MCHC at 0 and 4 h post-feeding are shown in Table 5. The values of glucose and PCV were 82.80 to 84.66 mg/dL and 30.66 to 31.83%, respectively. The glucose was in a normal range in goats, 50 to 75 mg/dL (2.77 to 4.16 mmol/L) [32]. Similar to Jain [33] who reported that the PCV values of the goat are in the range of 22 to 38%. The hematocrit is an essential marker for animal health to evaluate whether that animal has a blood disorder. When the PCV is lower than average, it indicates anemia. On the other hand, if the PCV is higher than average, the patient shows polycythemia symptoms, which are caused by abnormal red blood cell synthesis [33]. The triglyceride (TG), globulin, and MCV were affected by treatment, whereas TG concentration decreased in goat-fed increased EOPM diets. The globulin and MCV levels were found to be the highest in diets containing 30% EOPM. Castro et al. [16] found that increasing palm kernel oil from 0% to 5.2% DM in diets for finishing lambs increased serum cholesterol concentration. This may be due to the formation of very low-density lipids from saturated fatty acids in palm kernel oil used slowly by the extrahepatic tissues [34]. Similarly, Chanjula et al. [1] found that using palm kernel cake in concentrate for goats did not influence packed cell volume and glucose concentrations.

### 3.5. Volatile Fatty Acid Profile Concentration in the Rumen

Inclusions of EOPM in TMR diets did not change the VFA profiles (*p* > 0.05) except for TVFA concentration at 4 h post-feeding and mean values (Table 6). Feeding at 0 and 30% of EOPM had lower TVFAs than other groups and tended to linearly increase (L, *p* = 0.05 and 0.03, respectively) when EOPM levels were increased. Lunsin [2] used oil palm meal up to 30% DM in diets for lactating cows, resulting in a decrease in TVFAs and propionic acid concentration. This is possible as a result of the feed consumption and digestibility (OMI and NDFI), the digestible nutrients (NDF and ADF), and the feed intake chemical composition change among animals (Table 1). The TVFAs were increased in animals that received high levels of organic matter, intake of digestible nutrients of organic matter, and non-structural carbohydrates [23]. Furthermore, diets high in soluble carbohydrates and digestibility coefficients may increase propionic acid concentration in the rumen [35]. Chanjula et al. [1] found that TVFAs concentration was increased with 25% DM of palm kernel cake and decreased with more than 30% DM of palm kernel cake. Furthermore, depending on the kind of carbohydrate, it is affected by the quantity of total organic acids in the rumen and the amount ingested by the animals [36], roughage, and concentrate ratio [37]. Sutton et al. [38] indicated that the concentration of rapidly digested starch increased. This resulted in higher rumen propionic acid concentrations and lower acetic acid concentrations when comparing total volatile fatty acid (TVFAs) concentrations (TVFAs) and acid fractions. The TVFA_S_ tended to increase at the 4th h after feeding, consistent with Wanapat [39] reported that TVFAs increase and reach their peak 2 to 4 h after both morning and afternoon feedings. Based on the findings of this experiment, the mean total volatile fatty acid content of rumen fluid varied from 71.62 to 77.35 mmol/L. This was closely reported by Chanjula et al. [40], who found that the TVFAs of Anglo-Nubian 50% crossbreeds were 75.00 to 79.20 and 80.87 to 86.57 mmol/L, respectively, with the ruminal normal range as 70 to 130 mmol/L. Ruminal volatile fatty acid concentrations ranged from 70 to 150 mmol/L, which was linked to feeding consumption and organic matter coefficient digestibility [41]. Sutton [42] revealed that total volatile fatty acid synthesis is directly connected to OM digestibility. Additionally, acetic acid, butyric acid, and propionic acid concentrations were altered by the animal diet. Acetic acid is created in large volumes when the animal is fed a lot of roughage. According to Wanapat [43], the optimal rumen acetic acid, butyric acid, and propionic acid levels should be in the range of 65 to 70, 10 to 15, and 20 to 22% of TVFAs, with a C2:C3 ratio of 1 to 4, respectively. The ruminal acetic acid, butyric acid, and propionic acid concentrations should be 62, 16, and 22 % of TVFAs, respectively [28].

### 3.6. Rumen Microorganism Populations

The populations of ruminal bacteria and fungus were not affected by increasing EOPM inclusions, ranging from 1.49 to 1.75 × 10^10^ cells/mL and 1.52 to 2.29 × 10^6^ cell/mL, respectively (Table 7). This finding is consistent with Chanjula et al. [44] who reported that the bacterial and fungal populations of 50% Anglo-Nubian crossbred range from 1.40 to 2.23 × 10^10^ and 1.15 to 2.89 × 10^6^ cells/mL. Similarly, Hungate (1966) found that bacterial population and rumen fungus were in the range of 1.40 to 2.23 × 10^10^ and 1.15 to 2.89 × 10^6^ cells/mL, respectively. The protozoal population was not affected by EOPM inclusions, ranging from 2.41 to 2.99 × 10^6^ cells/mL. Hungate [45] also reported that the rumen protozoa population ranged from 10^4^ to 10^6^ cells/mL, but was lower than that reported by Chanjula et al. [44] who reported that the average protozoal population of hybrid goats was 10^4^ to 10^6^ cells/mL. Despite this, Khampa et al. [46] studies with castrated male dairy cows found that the protozoal population was 1.4 × 10^6^ cells/mL on average. Jouany and Ushida [47] reported that starch enrichment improved protozoal growth, which is supported by Russell [48] reporting that protozoal growth increased in the presence of starch. Furthermore, if the diet is starch-free, the density of protozoa and the rate of starch digestion are lowered. Moreover, the number of protozoa is affected by sugar. The decreasing protozoal population [47] had a positive effect on enhancing the bacterial population. Normally, protozoa thrive under environmental conditions and steal bacterial food. Russell [49] reported that the increased protozoal populations reduced the bacterial numbers, even though protozoa engulf bacteria as feed. In general, protozoa can consume up to 40% of the total number of bacteria ingested. However, if the diet contains a lot of whole grains and carbohydrates; protozoa can help to moderate the pH and prevent rumen acidity [50]. However, the rumen microbial population of each animal species depends on various factors, such as the type of feed, age, and rumen fermentation. Moreover, it was found that the high fiber diet had more cellulolytic bacteria than the low fiber diet. In addition, the level of NH_3_-N or digestibility performance by highly digestible diets and diets led to a surge in NH_3_-N levels in the rumen, leading to an increase in the number of bacteria [51].

### 3.7. Nitrogen Balance and Nitrogen Utilization

The total nitrogen balance intakes increased (*p* < 0.05) as the EOPM inclusions increased, possibly because the total feed intake increased with increasing EOPM levels in the diet, and protein digestibility tended to increase (*p* > 0.05) (Table 8). However, urinary N, fecal N, and total N excretion were unaffected by treatments (*p* > 0.05). Moreover, N absorption and nitrogen retention were not different (*p* > 0.05), but tended to increase with increased EOPM levels in the diet (*p* > 0.05). Despite this, the goat received correlated with the consumption of protein consumed individually and digestibility, with Absorbed N and Retained N averaging 67.42 to 68.81% and 48.35 to 53.78%, respectively. In addition, nitrogen balance and nitrogen utilization also increased in all goat groups. The use of EOPM rather than oil palm pulp (OPM) in the ready-mixed formula did not influence nitrogen balance. This might be because all the goats received nitrogen levels that were higher than their requirements. This was linked to the concentration of ammonia-nitrogen (NH_3_-N) in the rumen. They are higher than the optimal range for microbial development (5 to 8 mg/dL) [52] or 3.3 to 8.5 mg/100 mL [53].

## 4. Conclusions

Inclusions of EOPM in diets for goats increased feed intake, fiber digestibility, and blood parameters including globulin, triglyceride, and MCV concentration. The rumen parameters were not affected by the EOPM inclusions. Therefore, EOPM could be included in the diet for goats with up to 20% DM without negative effects. This study suggests that further studies should be conducted on the effect of EOPM on bigger numbers of animals to validate the present results.

## Figures and Tables

**Table 1:** Ingredients and chemical composition of goat diets formulated with various levels of extracted oil palm meal (EOPM) (% DM basis).

Item	Dietary Treatments				EOPM
	EOPM(0)	EOPM(10)	EOPM(20)	EOPM(30)	
Ingredients, % DM					
Low-fat oil palm meal	0.00	10.00	20.00	30.00	
Ground corn	44.50	35.00	25.43	15.78	
Soybean meal, (44% CP)	20.5	20.00	19.57	19.22	
Fish meal, 55% CP	0.50	0.50	0.50	0.50	
Leucaena leaves meal	5.00	5.00	5.00	5.00	
Oil palm fronds	25.00	25.00	25.00	25.00	
Molasses	3.00	3.00	3.00	3.00	
Salt	0.50	0.50	0.50	0.50	
Dicalcium phosphate	0.50	0.50	0.50	0.50	
Premix	0.50	0.50	0.50	0.50	
Total	100.0	100.0	100.0	100.0	
Chemical composition					
DM, %	89.50	90.40	91.20	92.10	95.86
OM, %DM	95.36	95.19	95.02	94.84	95.10
CP, %DM	15.27	15.26	15.27	15.31	10.62
EE, %DM	3.12	3.07	3.01	2.95	3.63
NDF, %DM	28.17	34.13	40.09	46.06	68.89
ADF, %DM	18.12	22.76	27.42	32.08	49.84
GE (kcal/kg DM)	4.61	4.53	4.32	3.57	4.95

EOPM(0) = 0% EOPM, EOPM(10) = 10% EOPM, EOPM(20) = 20% EOPM, EOPM(30) = 30% EOPM. DM = dry matter, OM = organic matter, CP = crude protein, EE = ether extract, NDF = neutral detergent fiber, ADF = acid detergent fiber, GE = gross energy. Premix (each kg contains): Vitamin A: 10,000,000 IU; Vitamin E: 70,000 IU; Vitamin D: 1,600,000 IU; Fe: 50 g; Zn: 40 g; Mn: 40 g; Co: 0.1 g; Cu: 10 g; Se: 0.1 g; I: 0.5 g.

**Table 2:** Feed intake of goats fed TMR diets containing extracted oil palm meal (EOPM).

Items	Dietary Treatments				SEM	*p*-Value	
	EOPM(0)	EOPM(10)	EOPM(20)	EOPM(30)		L	Q
Total DMI, kg/d	0.820 ^b^	0.867 ^ab^	0.890 ^a^	0.887 ^a^	0.02	0.01	0.14
DMI, %BW	2.56 ^b^	2.69 ^a^	2.73 ^a^	2.70 ^a^	0.03	0.01	0.03
DMI, g/kg W^0.75^	60.90 ^b^	64.14 ^a^	65.14 ^a^	64.44 ^b^	0.73	0.01	0.04
OMI, kg/d	0.78 ^b^	0.83 ^a^	0.84 ^a^	0.84 ^a^	0.02	0.02	0.07
CPI, kg/d	0.13	0.13	0.14	0.14	0.004	0.08	0.76
EEI, kg/d	0.03	0.03	0.03	0.03	0.002	0.41	0.53
NDFI, kg/d	0.23 ^b^	0.30 ^a^	0.34 ^a^	0.41 ^a^	0.008	0.001	0.08
ADFI, kg/d	0.15 ^b^	0.20 ^a^	0.24 ^a^	0.28 ^a^	0.005	0.001	0.30

^a–b^ Means within rows followed with different superscript letters are statistically different (*p* < 0.05). EOPM(0) = 0%. EOPM, EOPM(10) = 10% EOPM, EOPM(20) = 20% EOPM, EOPM(30) = 30% EOPM. DMI = dry matter intake, OMI = organic matter intake, CPI = crude protein intake, EEI = ether extract intake, NDFI = neutral detergent fiber intake, ADFI = acid detergent fiber intake. SEM = standard error of the mean. Orthogonal polynomial test for EOPM levels at L = linear and Q = quadratic.

**Table 3:** Nutrient digestibility and digestible nutrient intake of goats fed TMR diets containing extracted oil palm meal (EOPM).

Item	Dietary Treatments				SEM	*p*-Value	
	EOPM(0)	EOPM(10)	EOPM(20)	EOPM(30)		L	Q
Apparent total tract digestibility, %							
DM	57.66	58.69	59.15	59.36	0.59	0.08	0.51
OM	59.75	60.87	61.29	61.21	0.63	0.14	0.37
CP	66.69	68.79	69.21	69.50	1.25	0.16	0.49
EE	86.57	86.78	86.07	85.63	1.12	0.50	0.78
NDF	38.20 ^c^	40.61 ^bc^	45.35 ^a^	44.11 ^ab^	1.10	0.003	0.14
ADF	24.49 ^c^	28.43 ^b^	29.29 ^ab^	30.59 ^a^	0.55	0.001	0.05
Digestible nutrient intake, kg/d							
DOM	0.45 ^b^	0.48 ^ab^	0.50 ^a^	0.49 ^ab^	0.01	0.01	0.06
DCP	0.08 ^b^	0.09 ^a^	0.10 ^a^	0.10 ^a^	0.001	0.002	0.09
DEE	0.03	0.03	0.03	0.03	0.002	0.50	0.62
DNDF	0.15 ^c^	0.17 ^b^	0.21 ^a^	0.20 ^a^	0.006	0.001	0.03
DADF	0.06 ^c^	0.08 ^b^	0.08 ^ab^	0.09 ^a^	0.003	0.001	0.12
Estimated energy intake ^†^							
ME Mcal/d	1.72 ^b^	1.85 ^a^	1.90 ^a^	1.88 ^a^	0.04	0.01	0.07
ME Mcal/kg DM	2.10	2.13	2.14	2.12	0.02	0.37	0.30

^a–c^ Means within rows followed with different superscript letters are statistically different (*p* < 0.05). EOPM(0) = 0% EOPM, EOPM(10) = 10% EOPM, EOPM(20) = 20% EOPM, EOPM(30) = 30% EOPM. DM = dry matter, OM = organic matter, CP = crude protein, EE = ether extract, NDF = neutral detergent fiber, ADF = Acid detergent fiber, DOM = Organic matter digestibility, DCP = Crude protein digestibility, DEE = ether extract digestibility, DNDF = neutral detergent fiber digestibility, DADF = acid detergent fiber digestibility, ME = metabolizable energy. SEM = standard error of the mean. Orthogonal polynomial test for EOPM levels at L = linear and Q = quadratic. ^†^ 1 kg DOM = 3.8 Mcal ME/kg [11].

**Table 4:** Rumen fermentation of goats fed TMR diets containing extracted oil palm meal (EOPM).

Item	Dietary Treatments				SEM	*p*-Value	
	EOPM(0)	EOPM(10)	EOPM(20)	EOPM(30)		L	Q
Temperature, °C							
0 h post-feeding	39.20	39.30	39.00	39.10	0.15	0.62	0.67
4 h post-feeding	39.50	39.40	39.20	39.40	0.24	0.53	0.29
Mean	39.35	39.30	39.10	39.25	0.16	0.83	0.23
Ruminal pH							
0 h post-feeding	6.57	6.66	6.59	6.74	0.07	0.18	0.67
4 h post-feeding	6.26	6.35	6.37	6.31	0.06	0.53	0.27
Mean	6.42	6.50	6.49	6.53	0.05	0.27	0.72
NH_3_-N, mg/dL							
0 h post-feeding	19.64	20.00	18.57	19.29	2.05	0.79	0.93
4 h post-feeding	22.86	23.57	22.50	23.57	2.17	0.91	0.93
Mean	21.25	21.79	20.53	21.43	1.96	0.93	0.93
BUN, mg/dL							
0 h post-feeding	23.88	24.63	23.71	22.85	1.17	0.37	0.30
4 h post-feeding	23.94	24.46	25.03	23.20	0.86	0.52	0.09
Mean	23.91	24.55	24.37	23.03	0.99	0.42	0.18

EOPM(0) = 0% EOPM, EOPM(10) = 10% EOPM, EOPM(20) = 20% EOPM, EOPM(30) = 30% EOPM. NH_3_-N = ammonia nitrogen, BUN = blood urea nitrogen. SEM = standard error of the mean. Orthogonal polynomial test for EOPM levels at L = linear and Q = quadratic.

**Table 5:** Blood metabolites in goats fed TMR diets containing extracted oil palm meal (EOPM).

Item	Dietary Treatments				SEM	*p*-Value	
	EOPM(0)	EOPM(10)	EOPM(20)	EOPM(30)		L	Q
Glucose, mg/dL							
0 h post-feeding	58.75 ^b^	63.00 ^a^	64.00 ^a^	62.25 ^ab^	1.05	0.08	0.02
4 h post-feeding	67.00	64.25	64.00	63.75	1.15	0.11	0.32
Mean	62.87	63.62	64.00	63.00	0.84	1.00	0.34
PCV, %							
0 h post-feeding	31.75	31.50	30.00	31.00	0.62	0.22	0.35
4 h post-feeding	29.25	29.75	28.75	29.25	0.73	0.77	1.00
Mean	30.50	30.62	29.37	30.12	0.60	0.41	0.62
Cholesterol, mg%							
0 h post-feeding	129.50	126.50	126.00	116.00	4.73	0.14	0.21
4 h post-feeding	121.25	119.25	119.25	108.25	4.36	0.12	0.18
Mean	125.37	122.87	122.62	112.12	4.46	0.13	0.19
Triglyceride, mg%							
0 h post-feeding	52.75 ^a^	36.25 ^b^	35.50 ^b^	40.75 ^b^	2.96	0.03	0.01
4 h post-feeding	39.00	34.25	27.25	33.75	4.88	0.33	0.29
Mean	45.87 ^a^	35.25 ^b^	31.37 ^b^	37.25 ^ab^	2.91	0.06	0.03
Albumin, g%							
0 h post-feeding	4.11	3.90	3.96	4.05	0.07	0.74	0.09
4 h post-feeding	3.87	3.79	3.81	3.82	0.08	0.74	0.59
Mean	3.99	3.85	3.89	3.94	0.07	0.71	0.20
Globulin, g%							
0 h post-feeding	2.04	2.16	2.06	2.06	0.05	0.82	0.27
4 h post-feeding	2.04 ^b^	2.02 ^b^	2.04 ^b^	2.54 ^a^	0.06	0.001	0.01
Mean	2.04 ^b^	2.09 ^b^	2.05 ^b^	2.30 ^a^	0.03	0.01	0.02
WBC, 10^3^/µL							
0 h post-feeding	11.24	10.10	10.29	10.28	0.70	0.42	0.44
4 h post-feeding	11.48	11.12	10.90	11.08	0.76	0.68	0.73
Mean	11.37	10.62	10.60	10.68	0.69	0.53	0.57
RBC, 10^6^/µL							
0 h post-feeding	3.99	3.93	3.71	3.57	0.14	0.08	0.77
4 h post-feeding	3.61	3.67	3.43	3.50	0.13	0.35	0.95
Mean	3.80	3.80	3.57	3.54	0.11	0.08	0.89
HGB, g/dL							
0 h post-feeding	11.80	11.57	11.12	11.40	0.22	0.11	0.26
4 h post-feeding	10.87	10.75	10.50	10.62	0.26	0.43	0.65
Mean	11.33	11.16	10.81	11.01	0.19	0.18	0.38
MCV, fL							
0 h post-feeding	29.70 ^bc^	29.55 ^c^	30.22 ^a^	30.12 ^ab^	0.14	0.02	0.86
4 h post-feeding	30.15	30.12	30.90	30.42	0.26	0.22	0.42
Mean	29.92 ^b^	29.83 ^b^	30.56 ^a^	30.27 ^ab^	0.15	0.03	0.53
MCHC, g/dL							
0 h post-feeding	37.20	36.77	37.12	36.75	0.50	0.67	0.96
4 h post-feeding	37.15	37.12	36.57	36.32	0.64	0.33	0.86
Mean	37.17	36.95	36.85	36.53	0.38	0.28	0.91

^a–c^ Means within rows followed with different superscript letters are statistically different (*p* < 0.05). EOPM(0) = 0% EOPM, EOPM(10) = 10% EOPM, EOPM(20) = 20% EOPM, EOPM(30) = 30% EOPM. PCV = packed cell volume, WBC = white blood cells, RBC = red blood cells, HGB = hemoglobin, MCV = mean corpuscular volume, MCHC = mean corpuscular hemoglobin concentration. SEM = standard error of the mean. Orthogonal polynomial test for EOPM levels at L = linear and Q = quadratic.

**Table 6:** Volatile fatty acid profiles in goats fed TMR diets containing extracted oil palm meal (EOPM).

Item	Dietary Treatments				SEM	*p*-Value	
	EOPM(0)	EOPM(10)	EOPM(20)	EOPM(30)		L	Q
Total VFA, mmol/L							
0 h post-feeding	57.90	58.48	59.59	57.98	0.98	0.78	0.81
4 h post-feeding	87.37 ^b^	92.69 ^ab^	95.11 ^a^	87.58 ^b^	1.69	0.05	0.32
Mean	71.62 ^b^	75.58 ^a^	77.35 ^a^	72.78 ^b^	1.03	0.03	0.31
Proportion of individual VFA, %							
Acetate (C_2_)							
0 h post-feeding	71.16	70.66	69.38	71.21	0.65	0.29	0.51
4 h post-feeding	72.15	71.95	71.04	72.15	0.79	0.48	0.81
Mean	71.65	71.30	70.21	71.68	0.62	0.24	0.56
Propionate (C_3_)							
0 h post-feeding	19.61	19.26	20.21	19.61	0.51	0.83	0.76
4 h post-feeding	20.75	20.19	21.50	20.75	0.58	0.67	0.31
Mean	20.17	19.73	20.85	20.17	0.47	0.69	0.42
Butyrate (C_4_)							
0 h post-feeding	6.06 ^b^	7.24 ^a^	7.51 ^a^	6.06 ^b^	0.34	0.16	0.62
4 h post-feeding	5.22	6.01	5.63	5.22	0.54	0.35	0.87
Mean	5.64	6.63	6.57	5.63	0.37	0.16	0.72
Other VFA^⸹^							
0 h post-feeding	3.22	2.83	2.84	3.21	0.07	0.28	0.94
4 h post-feeding	1.88	1.83	1.82	1.88	0.11	0.09	0.29
Mean	2.54	2.33	2.33	2.54	0.09	0.10	0.65
Acetate: propionate ratio							
0 h post-feeding	1.74	1.57	1.76	1.63	0.08	0.72	0.84
4 h post-feeding	1.82	1.91	1.77	1.63	0.11	0.20	0.36
Mean	1.78	1.73	1.77	1.63	0.08	0.31	0.62
Acetate, butyrate: propionate ratio							
0 h post-feeding	2.25	1.97	2.21	2.03	0.15	0.57	0.74
4 h post-feeding	2.28	2.39	2.25	2.09	0.16	0.37	0.42
Mean	2.26	2.18	2.23	2.06	0.12	0.35	0.72
CH_4_, mol %							
0 h post-feeding	22.28	20.34	22.12	21.00	1.02	0.66	0.70
4 h post-feeding	22.67	23.39	22.22	21.27	1.01	0.28	0.43
Mean	22.48	21.87	22.17	21.13	0.84	0.36	0.80

^a,b^ Means within rows followed with different superscript letters are statistically different (*p* < 0.05). EOPM(0) = 0% EOPM, EOPM(10) = 10% EOPM, EOPM(20) = 20% EOPM, EOPM(30) = 30% EOPM. SEM = standard error of the mean, VFA = volatile fatty acids, CH_4_ = methane. Orthogonal polynomial test for EOPM levels at L = linear and Q = quadratic. ^⸹^Sum of isobutyrate, isovalerate, valerate and caproate.

**Table 7:** Rumen microbes in goats fed TMR diets containing extracted oil palm meal (EOPM).

Item	Dietary Treatments				SEM	*p*-Value	
	EOPM(0)	EOPM(10)	EOPM(20)	EOPM(30)		L	Q
Total direct count							
Bacteria (×10^10^ cell/mL)							
0 h post-feeding	1.45	1.45	1.60	1.35	1.35	0.50	0.67
4 h post-feeding	1.56	1.67	1.90	1.63	2.01	0.67	0.80
Mean	1.51	1.56	1.75	1.49	1.65	0.43	0.89
Fungal zoospores (×10^6^ cell/mL)							
0 h post-feeding	1.53	1.61	1.91	1.67	0.27	0.07	0.51
4 h post-feeding	1.52	1.51	2.67	2.15	0.37	0.11	0.72
Mean	1.52	1.56	2.29	1.91	0.28	0.06	0.97
Total Protozoa (×10^6^ cell/mL)							
0 h post-feeding	2.29	2.47	2.21	2.51	0.26	0.09	0.50
4 h post-feeding	2.61	3.15	2.63	3.47	0.32	0.10	0.56
Mean	2.46	2.81	2.41	2.99	0.26	0.06	0.95

EOPM(0) = 0% EOPM, EOPM(10) = 10% EOPM, EOPM(20) = 20% EOPM, EOPM(30) = 30% EOPM. SEM = standard error of the mean. Orthogonal polynomial test for EOPM levels at L = linear and Q = quadratic.

**Table 8:** N balance of goats fed TMR diets containing extracted oil palm meal (EOPM).

Item	Dietary Treatments				SEM	*p*-Value	
	EOPM(0)	EOPM(10)	EOPM(20)	EOPM(30)		L	Q
N balance, g/d							
Total N intake	21.54 ^c^	21.97 ^bc^	23.16 ^ab^	23.49 ^a^	0.47	0.01	0.89
N excretion, g/d							
Fecal N	7.05	7.24	7.25	7.47	0.48	0.57	0.98
Urinary N	4.12	3.42	3.50	4.08	0.74	0.99	0.42
Total N excretion	11.17	10.66	10.76	11.55	1.12	0.81	0.58
Absorbed N	14.48	14.73	15.90	16.02	0.51	0.10	0.87
Retained N	10.36	11.31	12.40	11.93	0.98	0.23	0.50
N output (% of N intake)							
Absorbed	67.42	66.99	68.92	68.81	1.90	0.50	0.93
Retained	48.35	51.08	53.78	52.54	4.73	0.49	0.68

^a–c^ Means within rows followed with different superscript letters are statistically different (*p* < 0.05). EOPM(0) = 0% EOPM, EOPM(10) = 10% EOPM, EOPM(20) = 20% EOPM, EOPM(30) = 30% EOPM. SEM = standard error of the mean. Orthogonal polynomial test for EOPM levels at L = linear and Q = quadratic.

## Data Availability

Not applicable.
